# PCSK9 inhibition alleviates sepsis-induced myocardial dysfunction by facilitating PINK1/parkin-associated mitophagy

**DOI:** 10.3389/fphar.2026.1844269

**Published:** 2026-07-03

**Authors:** Jiancheng Lin, Zetao Pan, Jiayan Sun, Xiaowan Wang, Linna Zhang, Yuheng Ye, Di Yin, Yi Wang, Qiang Guo

**Affiliations:** 1 Medical College of Soochow University, Suzhou, Jiangsu, China; 2 Medical Center of Soochow University, Suzhou, Jiangsu, China; 3 Department of Emergency and Critical Care Medicine, The Fourth Affiliated Hospital of Soochow University (Suzhou Dushu Lake Hospital), Suzhou, Jiangsu, China; 4 Department of Critical Care Medicine, The Affiliated Hospital of Xuzhou Medical University, Xuzhou, Jiangsu, China

**Keywords:** evolocumab, mitochondrial dynamics, mitochondrial quality control, mitophagy, PCSK9, sepsis-induced myocardial dysfunction

## Abstract

**Introduction:**

Sepsis-induced myocardial dysfunction (SIMD), also known as septic cardiomyopathy in sepsis patients is associated with worse prognosis and higher mortality compared to sepsis cases without SIMD. Early intervention and comprehensive management are crucial for improving survival, particularly in the early stages of sepsis. Proprotein convertase subtilisin/kexin type 9 (PCSK9) is a promising therapeutic target in the cardiovascular system. While PCSK9 has been implicated in cardiovascular inflammation and injury, specific evidence regarding the potential of PCSK9 inhibition to mitigate SIMD remains limited.

**Methods:**

An *in vivo* mouse model of sepsis was established using cecal ligation and puncture (CLP), and SIMD was assessed through echocardiography and right ventricular systolic pressure measurements. For *in vitro* cellular SIMD models, lipopolysaccharide was administered to HL-1 and H9c2 cardiomyocytes. Data concerning apoptosis, inflammatory responses, oxidative stress, and mitophagy were evaluated across both models.

**Results:**

The SIMD models were successfully established both *in vivo* and *in vitro*. PCSK9 inhibition visibly attenuated myocardial dysfunction, cellular injury, apoptosis, inflammation, and oxidative stress, which was accompanied by enhanced mitophagic clearance. Furthermore, the application of Mdivi-1, a mitochondrial division inhibitor that impairs mitophagy, revealed that the cardioprotective effects of PCSK9 inhibition are at least partially dependent on the preservation of mitophagy. Mechanistically, PCSK9 inhibition appeared to facilitate mitophagic flux in SIMD, potentially via the PINK1/Parkin signaling pathway.

**Conclusion:**

These findings suggest that the protective effects of PCSK9 inhibition against SIMD are closely associated with the enhancement of mitophagy and the modulation of the PINK1/Parkin pathway. Furthermore, this intervention correlates with attenuated oxidative stress, inflammation, and apoptosis, ultimately offering a potential therapeutic strategy for myocardial injury.

## Graphical Abstract

**Figure:**
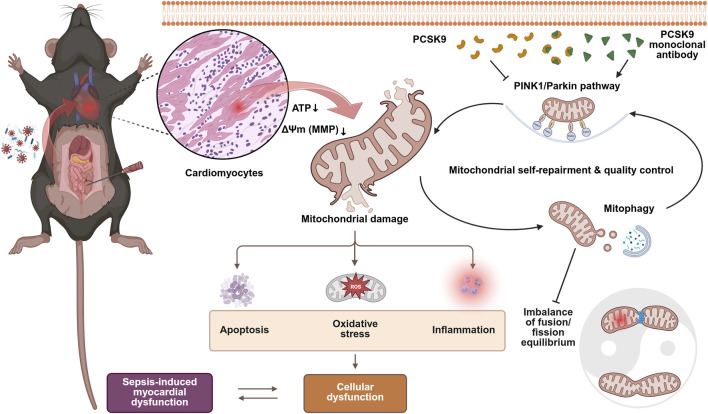

## 1 Introduction

Sepsis is a life-threatening syndrome that occurs when the host’s response to infection triggers widespread inflammation, resulting in tissue damage, organ failure, and potentially death (Singer et al., 2026). The heart, as a critical target organ in sepsis, plays a crucial role in the pathophysiology of sepsis (Aissaoui et al., 2025). In cases where the infection progresses to septic shock, cardiovascular dysfunction is often the primary cause of death, primarily due to the inability to maintain adequate tissue oxygenation (Hiraiwa et al., 2024). Sepsis-induced myocardial dysfunction (SIMD), also known as septic cardiomyopathy is a major contributor to this situation, accounting for its reversible form of global cardiac disorder (Hollenberg and Singer, 2021).

Mitochondria, as primary energy providers, are extensively distributed across nearly all cell types, particularly within the heart. Cardiomyocytes are densely populated with mitochondria, which can account for up to 30%–40% of the cell’s volume, reflecting their significant energy demands (Ravindran and Rau, 2024). Additionally, mitochondria are crucial targets in SIMD due to their central roles in cellular energy production (Stanzani et al., 2019), as well as in the regulation of inflammatory responses, cellular apoptosis, and oxidative stress (Zhang et al., 2022; Galley, 2011; Yan et al., 2024). Therefore, maintaining mitochondrial function is of paramount importance (Hu et al., 2024).

Mitochondrial quality control is a critical mechanism that maintains mitochondrial health and function (Yang and Zhang, 2021). This process encompasses a network of complex pathways, including mitophagy, a specialized form of autophagy, as well as mitochondrial dynamics, mitochondrial DNA (mtDNA) repair, and mitochondrial biogenesis (Kuroshima et al., 2024). Among these, mitophagy is considered a hallmark (Mohsin et al., 2021). The PTEN-induced kinase 1 (PINK1) and Parkin signaling pathway, one of the most well-studied in this context, has been shown to play a protective role in the heart when appropriately activated (Zhao et al., 2024).

Proprotein convertase subtilisin/kexin type 9 (PCSK9), a serine protease, plays a critical role in cholesterol metabolism, particularly through its regulation of low-density lipoprotein receptors (LDLRs) on hepatocytes (McPherson et al., 2024). In addition to its established function in lipid metabolism, PCSK9 has been increasingly recognized for its involvement in other biological processes, including inflammation, apoptosis, and oxidative stress—key pathophysiological features of sepsis (Seidah and Prat, 2022; Xu et al., 2023; Park et al., 2024). Furthermore, recent studies have highlighted the connection among PCSK9, mitochondria quality control and cardiac protection, providing valuable insights into mitochondrial quality control mechanisms in cardiovascular and metabolic diseases (Cao et al., 2023).

Given the limited research on the association between PCSK9, SIMD and mitochondrial quality control, we hypothesize that PCSK9 exacerbates SIMD by impairing mitochondrial mitophagy, and that its pharmacological inhibition can alleviate cardiac injury by restoring mitochondrial quality control. Additionally, we seek to identify potential therapeutic targets for SIMD and provide experimental evidence to support it.

## 2 Materials and methods

### 2.1 Reagents and chemicals

Major reagents and chemicals utilized in this study included phosphate-buffered saline (PBS), dimethyl sulfoxide (DMSO), lipopolysaccharide (LPS), evolocumab (EVO), mitochondrial division inhibitor (Mdivi-1), and chloroquine phosphate (CQ). LPS and Mdivi-1 were purchased from Selleck Chemicals (Houston, TX, USA). EVO was obtained from Amgen Inc. (Thousand Oaks, CA, USA). CQ, an autophagy inhibitor, was purchased from Beyotime Biotechnology (Shanghai, China). PBS and DMSO vehicle were both obtained from Solarbio Science and Technology Co., Ltd. (Beijing, China).

### 2.2 Experimental animals

Eight-week-old C57BL/6J mice, obtained from Ziyuan Laboratory Animal Technology Co., Ltd. (Zhejiang, China), were housed under standard laboratory conditions (25 °C ± 2 °C, 53% ± 3% humidity, and a 12-h light-dark cycle) with *ad libitum* access to a standard diet and water. After a 1-week acclimatization period, mice weighing 23 ± 2g were randomly allocated into experimental groups. Three independent cohorts (with an initial allocation of n = 10 per group to account for potential CLP-induced mortality) were designed as follows: (1) to observe whether PCSK9 levels differ in SIMD, mice were assigned into Sham and CLP groups; (2) to assess whether a PCSK9 inhibitor could exert an impact on sepsis-induced cardiac dysfunction, myocardial injury, inflammatory reactions, cellular apoptosis, oxidative stress, and mitochondrial dynamics, mice were divided into Sham, CLP, and EVO groups; (3) to evaluate whether mitophagy serves as a key hub between SIMD and other phenotypes, mice were separated into Sham, CLP, EVO, and Mdivi-1 groups. Each independent cohort included its own concurrent Sham and CLP groups to strictly preclude the reuse of baseline controls and avoid batch effects. To minimize selection bias, the randomization was performed by an independent investigator using a computer-generated random number sequence. All subsequent procedures and data analyses were conducted by researchers blinded to the group assignments. The final number of biologically independent samples successfully analyzed (n = 6 per group) is explicitly detailed in the respective figure legends. The timeline of the experimental procedures is illustrated in Figure 2A. Briefly, following the 7-day adaption period, the SIMD model was established via cecal ligation and puncture (CLP) surgery on Day 7. Thirty minutes post-surgery, pharmacological interventions were administered. At 24 h post-CLP or Sham operation, cardiac function was evaluated via echocardiography and right ventricular systolic pressure (RVSP) measurement, immediately after which the mice were euthanized for tissue and blood sample collection.

All animal experiments were designed and reported in strict accordance with the ARRIVE guidelines. The experimental protocols were reviewed and approved by the Ethics Committee of The Fourth Affiliated Hospital of Soochow University (Suzhou Dushu Lake Hospital) (Approval No. 241009), and all procedures were conducted following the Guide for the Care and Use of Laboratory Animals.

### 2.3 Cecal ligation and puncture (CLP) procedure and treatment

All mice were anesthetized with 3%–4% isoflurane for induction and maintained with 1%–2% isoflurane during the procedure. The abdominal fur from the xiphoid process to the pubis was shaved, and a laparotomy was performed using a sterile scalpel. Then mice in Sham group only had cecum fully exposed while mice in CLP, EVO and Mdivi-1 group had cecum carefully exteriorized by sterile hemostats and forceps before ligation and penetration with 4–0 silk suture and 22-gauge needle respectively. A small amount of fecal matter was gently squeezed to extrude from the puncture site. After correctly repositioning the cecum without twisting or torsion, all mice’s peritoneal layer and skin incision were closed by 5–0 nylon sutures.

Following 30 min of postoperative care and monitoring, EVO group was intraperitoneally administered EVO (50 mg/kg). Mdivi-1 group received intraperitoneal injection of EVO (50 mg/kg) and Mdivi-1 (10 mg/kg). Equal amount of saline was intraperitoneally administered to mice in Sham and CLP group.

### 2.4 Survival analysis

To evaluate the effects of the respective treatments on the survival rate of CLP mice, separate cohorts of mice (n = 20 per group) were assigned using the aforementioned randomization protocol. Following the CLP procedure and respective pharmacological treatments, the general condition and survival status of the mice were closely monitored and recorded every 12 h over a 7-day observation period. To adhere to ethical standards and minimize animal suffering, strictly defined humane endpoints were applied throughout the study. Mice exhibiting signs of irreversible moribundity, such as a complete loss of righting reflex, severe hypothermia, unresponsiveness to external stimuli, or a prolonged inability to access food and water were promptly euthanized and recorded as mortalities in the survival analysis.

### 2.5 Echocardiography

Echocardiographic examination was conducted by an ultrasound system (VINNO 6 LAB, Suzhou, China) after 24 h from surgical procedure. Mice in all groups were anesthetized with 3%–4% isoflurane for induction and maintained at 1%–2% isoflurane. They were then secured on a platform and examined using a 23-MHz linear transducer. Data acquisition was performed using M-mode guided by two-dimensional imaging from the parasternal short-axis view at the papillary muscle level. Left ventricular ejection fraction (LVEF), left ventricular fraction shortening (LVFS), left ventricular internal diameter in diastole (LVIDd) and systole (LVIDs) were captured and measured, with all parameters calculated by averaging the measurements from three consecutive cardiac cycles, to evaluate left cardiac structure and function in mice. If myocardial dysfunction was indicated in mice from CLP, EVO and Mdivi-1 group by echocardiography, they continued to get their right ventricular function detected; if not, they were euthanized by cervical dislocation.

### 2.6 RVSP measurement

Under ventilator anesthesia, RVSP was detected using a microtip on Millar pressure transducer catheter (SPR-839, ADInstruments, Australia), which was inserted into mice right ventricle via the right external jugular vein. The curve graph depicting RVSP was continuously recorded and subsequently analyzed with the PowerLab system and LabChart software to assess mice right ventricular hemodynamics. After the data acquirement, all mice were respectably sacrificed by cervical dislocation, then blood and heart tissues were collected.

### 2.7 Histopathology

The collected heart tissues were fixed in 4% paraformaldehyde (PFA) for about 24 h and subsequently embedded in paraffin before sectioning into 5 μm slices. These sections then underwent histopathological staining using hematoxylin-eosin (H&E) stain kit (Servicebio, China).

### 2.8 Histological immunofluorescence

Following deparaffinization and rehydration, heart tissue sections were treated with 3% hydrogen peroxide for 20 min to quench endogenous peroxidase activity, and subsequently blocked with 5% serum containing 0.4% Triton X-100 for 2 h at room temperature. The sections were then incubated with the corresponding primary antibodies overnight at 4 °C. After rigorous washing with PBS, sections were incubated with the appropriate secondary antibodies for 1 h at room temperature. Detailed information regarding all primary and secondary antibodies, including specific dilution ratios, is comprehensively summarized in Supplementary Table S1. Finally, the resulting images were captured using an ECLIPSE Ti2 inverted fluorescent microscope (standard widefield epifluorescence mode, Nikon, Japan).

### 2.9 Cell culture and treatment

Mouse cardiomyocyte (HL-1) and rat cardiomyocyte (H9c2) cell line were obtained from the National Collection of Authenticated Cell Cultures (Shanghai, China) and maintained in DMEM/F12 cell culture medium (Procell, China) supplemented with 10% fetal bovine serum (FBS) (ABW, China). The cells were cultured in a humidified atmosphere at 37 °C with 5% CO2. Cell counting kit-8 (Beyotime, China) was applied to detect HL-1 cells vitality in different groups.

### 2.10 Cellular immunofluorescence

HL-1 cells were cultured in confocal dishes and fixed with 4% PFA for half an hour before 2 h permeabilization using 0.1% Triton X-100 at room temperature. Then primary antibodies including TOM20 and LAMP1 (Proteintech, China), respectively labeling mitochondria and lysosomes, were used for hatching with HL-1 cells overnight at 4 °C. Detailed information regarding both antibodies, including their specific dilution ratios, is summarized in Supplementary Table S1. After incubation with cognate secondary antibody, the relative movement status between cellular mitochondria and lysosomes were captured by an ECLIPSE Ti2 inverted fluorescent microscope (confocal laser scanning imaging mode, Nikon, Japan).

### 2.11 Apoptosis detection

The apoptotic processes of mice heart tissues and HL-1 cells were respectively tested by TdT-mediated dUTP nick-end labeling (TUNEL) assay kit (Beyotime, China) and immunofluorescence incubating with cleaved-Caspase 3 (Proteintech, China) antibody according to the manufacturer protocol. Detailed antibody’s information is summarized in Supplementary Table S1. Resulting images were captured by an ECLIPSE Ti2 inverted fluorescent microscope (standard widefield epifluorescence mode, Nikon, Japan).

### 2.12 Colorimetric assay

Mouse serum, heart tissue homogenates, and HL-1 cell supernatants were collected to measure the levels of tumor necrosis factor-α (TNF-α), interleukin-1β (IL-1β), interleukin-6 (IL-6), cardiac troponin I (cTnI), and creatine kinase muscle-brain (CK-MB) using corresponding enzyme-linked immunosorbent assay (ELISA) and biochemical kits (Servicebio, China) according to the manufacturers’ instructions. Furthermore, oxidative stress parameters, including superoxide dismutase (SOD), glutathione (GSH), and malondialdehyde (MDA), along with adenosine triphosphate (ATP) content, were measured using specific assay kits (Nanjing Jiancheng Bioengineering Institute, Nanjing, China) following their respective product manuals. All resulting optical densities were captured and analyzed using a Multiskan™ FC Microplate Photometer (Thermo Fisher Scientific, US).

### 2.13 Reactive oxygen species (ROS) and mitochondrial membrane potential (MMP, ΔΨm) assay

For frozen mice heart tissue sections, ROS was detected with ROS assay kit (Beyotime, S0033S). For cellular experiments, HL-1 cells were cultured in 6-well plates before incubation with DCFH-DA and JC-1 (Solarbio, M8650) at 37 °C for 30 min. Ultimately, ROS content and MMP were measured by a Multiskan™ FC multifunctional microplate photometer (Thermo Fisher Scientific, US), an ECLIPSE Ti2 inverted fluorescent microscope (standard widefield epifluorescence mode, Nikon, Japan) or a CytoFLEX S flow cytometer (Beckman Coulter, US).

### 2.14 Transmission electron microscopy (TEM)

To observe the ultrastructural pathology both *in vivo* and *in vitro*, mice heart tissues and HL-1 cells were fixed in 2.5% glutaraldehyde and subsequently dehydrated using concentration-stagged alcohol and propanone. After embedding in epoxy resin, 70 nm ultrathin sections were sliced and double-stained by 2% uranyl acetate and lead citrate. Afterwards, images of cellular ultrastructure were captured by a transmission electron microscope (Hitachi, Japan).

### 2.15 DNA isolation and real-time polymerase chain reaction (qPCR)

Genomic DNA (gDNA) was isolated and purified from mouse heart samples and HL-1 cells using the TIANamp DNA Kit (TIANGEN, DP304) following the manufacturer’s protocol. This procedure aimed to determine the ratio of mitochondrial DNA (mtDNA) to nuclear DNA (nDNA) as well as the mtDNA copy number according to previous researches (Quiros et al., 2017; Bueno et al., 2015). Quantitative PCR (qPCR) was then performed to amplify one gene from the mouse mitochondrial genome (*Nd2*) and one gene from the nuclear genome (*Gapdh*). The primer sequences used were as follows.

- *Nd2* forward: 5’ - CCC​ATT​CCA​CTT​CTG​ATT​ACC - 3′
- *Nd2* reverse: 5’ - ATG​ATA​GTA​GAG​TTG​AGT​AGC​G - 3′
- *Gapdh* forward: 5’ – CCT​GCA​CCA​CCA​ACT​GCT​TAG - 3′
- *Gapdh* reverse: 5’ – GTG​GAT​GCA​GGG​ATG​ATG​TTC - 3′

### 2.16 Western blot

Mice heart tissues and cultured cells (HL-1 and H9c2) were lysed using lysis buffer (Thermo Fisher Scientific, US) supplemented with protease and phosphatase inhibitor cocktails. Protein concentrations were measured using a BCA protein assay kit (Beyotime, P0010S). Equal amounts of protein (10–20 μg) were separated by SDS-PAGE (8%–12%) and transferred onto Millipore polyvinylidene fluoride (PVDF) membranes (Merck, Germany). After blocking with Quick Blocking Buffer (Beyotime Biotechnology, Shanghai, China) for 60 min at room temperature, PVDF membranes were incubated with primary antibodies against PCSK9, Drp1, Mfn2, LC3, SQSTM1, TOM20, TIM23, PINK1, and Parkin (Proteintech, China) overnight at 4 °C. After rigorous washing, the membranes were incubated with the cognate secondary antibodies for 1 h at room temperature. Detailed information regarding all antibodies, including their specific dilution ratios, is summarized in Supplementary Table S1. Protein bands were visualized using ECL detection reagents in a fluorescence imaging analysis system (Sinsage, China) and quantified with ImageJ software (V1.8.0.172). The relative protein expression levels were determined by calculating the ratio of the target protein to the corresponding housekeeping protein.

### 2.17 Statistical analysis

Data from *in vitro* studies were obtained from a minimum of three independent experiments (n ≥ 3) and analyzed using GraphPad Prism software (V9.50, California, US). All data are presented as the mean ± standard deviation (SD). The sample sizes for *in vivo* animal studies (n = 6 per group) were determined based on our preliminary experimental data, comparable prior literature, and the 3R principles of animal welfare to minimize animal use while ensuring sufficient statistical power. Before applying parametric tests, the normal distribution of the datasets was confirmed using the Shapiro-Wilk test, and the homogeneity of variances was evaluated. Differences between two groups were assessed using Student’s t-test with Welch’s correction to account for potentially unequal variances. Additionally, for relative quantitative data expressed as fold changes, where the control group was normalized to a baseline constant of 1, a one-sample t-test was employed to compare the experimental group against the theoretical mean of 1.0. For multiple comparisons among three or more groups, one-way analysis of variance (ANOVA) followed by the Bonferroni *post hoc* test was employed. A P-value <0.05 was considered statistically significant.

## 3 Results

### 3.1 The PCSK9 expression is increased in the heart and serum of mice with SIMD

**FIGURE 1:**
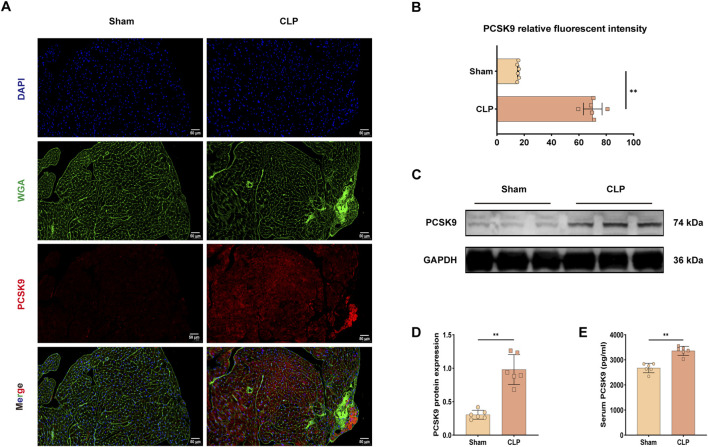
PCSK9 expression in hearts and serum are increased in cecal ligation and puncture (CLP) mice. **(A)** Representative immunofluorescent images co-stained with PCSK9 and WGA of heart tissue from CLP mice (scale bar = 50 μm, magnification: ×20). **(B)** Statistical assessment of PCSK9 relative fluorescent intensity. **(C)** Representative Western blot images of PCSK9 in heart tissue of mice with CLP. **(D)** Quantitative analysis of PCSK9 expression in Western blot images. **(E)** Quantification of serum PCSK9 levels in CLP mice by colorimetry. N = 6 per group. **, p < 0.01 versus Sham group.

To investigate whether PCSK9 is a potential therapeutic target in SIMD, we assessed the expression of PCSK9 and wheat germ agglutinin (WGA) through immunofluorescence analysis. The results (Figures 1A,B) revealed an abnormal accumulation and aggregation of WGA on the cardiomyocyte membrane, a marked enlargement in cardiomyocyte size, and a significant increase in PCSK9 immunofluorescence intensity, particularly in the enlarged regions, in the CLP group. Additionally, Western blotting and ELISA were conducted to evaluate the changes in PCSK9 expression levels in heart tissue and serum. As shown in Figures 1C–E, PCSK9 expression was significantly elevated in both heart tissue and serum samples. These findings indicate that the CLP operation employed in this study effectively induces myocardial tissue alterations and that elevated PCSK9 levels are closely associated with SIMD.

### 3.2 PCSK9 inhibitor alleviates global cardiac dysfunction in mice with SIMD

**FIGURE 2:**
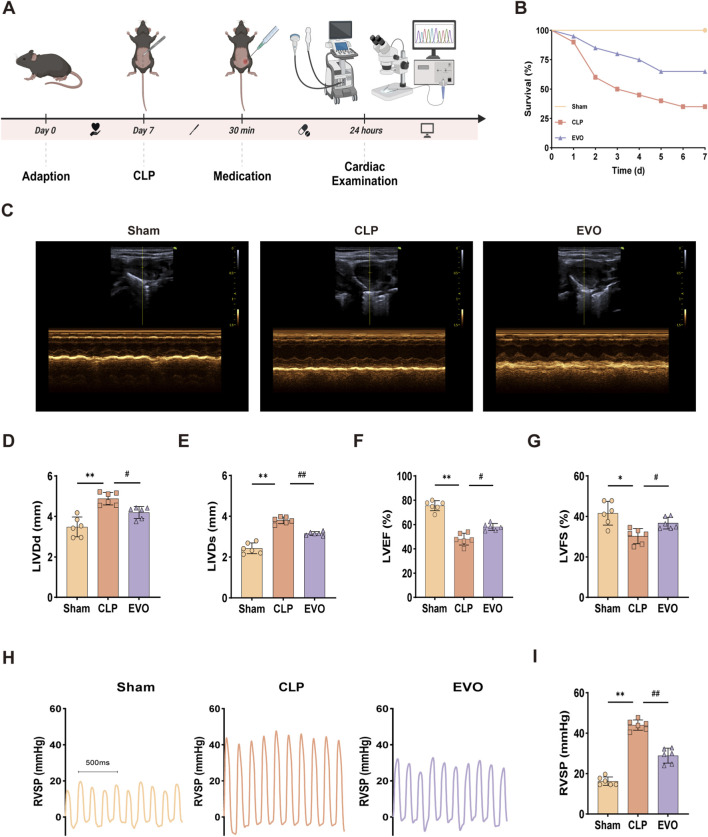
PCSK9 inhibitor alleviates CLP-induced heart dysfunction in mice. **(A)** Experimental timeline and workflow diagram (created with BioRender.com) **(B)** 7-day survival rates of mice post-CLP across groups (n = 20 per group). **(C)** Representative echocardiographic snapshots of the left ventricle. **(D)** Quantification of left ventricular internal diameter in diastole (LVIDd) **(E)** left ventricular internal diameter in systole (LVIDs) **(F)** left ventricular ejection fraction (LVEF) and **(G)** left ventricular fraction shortening (LVFS) from ultrasound images. **(H)** Typical right ventricular systolic pressure (RVSP) curve. **(I)** Quantification of RVSP. n = 6 per group. *, p < 0.05 and **, p < 0.01 versus Sham group. #, p < 0.05 and ##, p < 0.01 versus CLP group.

Further investigation into the role of PCSK9 was conducted according to the timeline presented in Figure 2A. The survival curve analysis (Figure 2B) showed that the survival rate was significantly higher in the EVO group compared to the CLP group.

Echocardiographic assessment of left ventricular function revealed that mice in the CLP group exhibited left ventricular dysfunction. However, inhibition of PCSK9 led to strengthened EF and FS, which subsequently alleviating left ventricular dysfunction (Figures 2C–G). Additionally, the right ventricular function was assessed by measuring the RVSP following echocardiography. The results (Figures 2H,I) indicated that RVSP was significantly elevated after CLP surgery but was markedly reduced in the EVO group. These findings suggest that the CLP model induces left ventricular structural alterations and imposes a significant hemodynamic burden on the right ventricle, both of which are alleviated by PCSK9 inhibition.

### 3.3 PCSK9 inhibitor ameliorates cardiac injury, inflammatory response and cell apoptosis of heart in mice with SIMD

**FIGURE 3:**
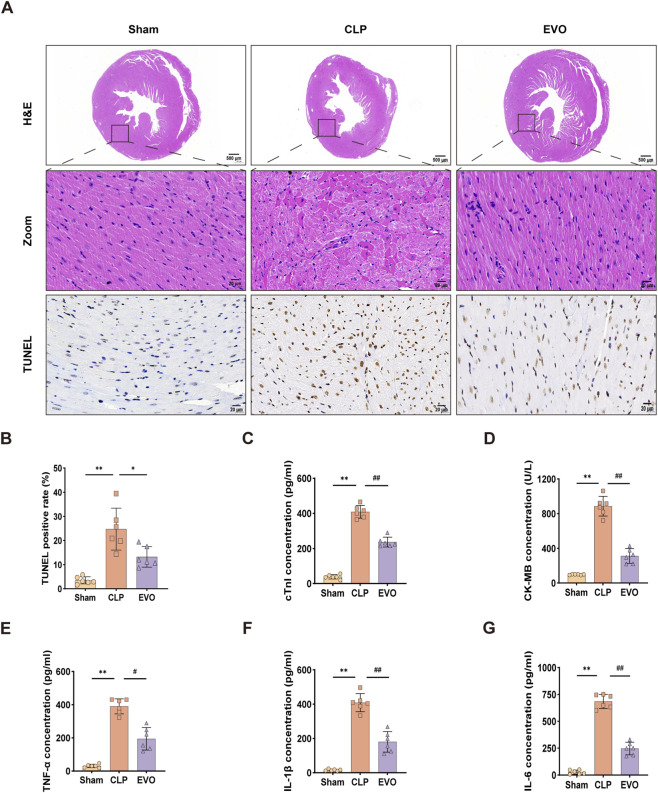
PCSK9 inhibitor ameliorates cardiac injury, apoptosis and inflammation in mice with sepsis-induced myocardial dysfunction (SIMD). **(A)** Representative histopathological structure (scale bar = 500 μm, magnification: ×2, zoomed scale bar = 20 μm, zoomed magnification: ×40) and TUNEL immunohistochemical images (scale bar = 20 μm, magnification: ×40) of mice hearts. **(B)** Quantitative analysis of positive rate of TUNEL immunohistochemistry. **(C)** Serum concentration of cardiac troponin I (cTnI) **(D)** creatine kinase muscle-brain (CK-MB) **(E)** tumor necrosis factor-α (TNF-α) **(F)** interleukin-1β (IL-1β) and **(G)** interleukin-6 (IL-6) were detected via colorimetric assay. N = 6 per group. *, p < 0.05 and **, p < 0.01 versus Sham group. #, p < 0.05 and ##, p < 0.01 versus CLP group.

Histopathological alterations were further assessed by staining heart tissue sections with H&E and performing TUNEL assays. As shown in Figures 3A,B, myocardial fibers in the CLP group were disorganized, with edema and infiltration of inflammatory cells. In contrast, the heart tissue condition in the EVO group showed significant improvement, resembling that of the Sham group. Meanwhile, consistent with the histopathological changes, the TUNEL staining positivity rate was increased in the CLP group and decreased in the EVO group, indicating that inflammation and cell apoptosis occurred simultaneously. Additionally, the levels of typical pro-inflammatory cytokines and myocardial injury biomarkers were measured to further assess the inflammatory phenotype and myocardial lesions. The results (Figures 3C–G) revealed that, following CLP, levels of TNF-α, IL-1β, and IL-6 were significantly elevated, with similar trends observed for cTnI and CK-MB. These effects were attenuated in the EVO group. Collectively, these findings indicate that PCSK9 inhibition by EVO effectively alleviates structural myocardial injury, suppresses the inflammatory response, and reduces cardiomyocyte apoptosis in SIMD.

### 3.4 PCSK9 inhibitor attenuates oxidative stress, mitochondrial damage and enhances mitochondrial dynamics in mice with SIMD

**FIGURE 4:**
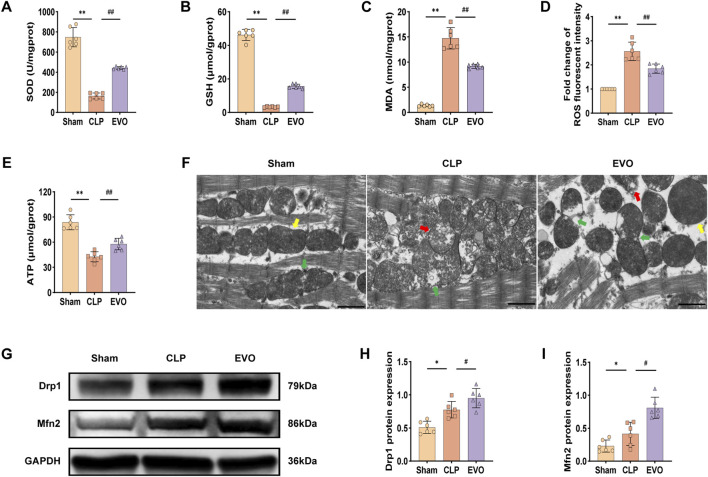
PCSK9 inhibitor attenuates oxidative stress and the imbalance between mitochondrial damage and mitochondrial dynamics in SIMD mice heart. **(A)** Quantification of superoxide dismutase (SOD) **(B)** glutathione (GSH) **(C)** malondialdehyde (MDA) **(D)** reactive oxygen species (ROS) and **(E)** adenosine triphosphate (ATP) in heart tissue were measured using colorimetric analysis. **(F)** Representative snapshots of cardiac TEM (scale bar = 1 μm, magnification: 10000×), yellow arrows indicate normal mitochondria, red arrows denote abnormal mitochondria (e.g., swollen with disrupted cristae), and green arrows represent mitochondrial dynamics (fission or fusion). **(G)** Illustrative Western blot images of Drp1 and Mfn2 in heart. **(H)** Statistical analysis of Drp1 and **(I)** Mfn2 of Western blot images. N = 6 per group. *, p < 0.05 and **, p < 0.01 versus Sham group. #, p < 0.05 and ##, p < 0.01 versus CLP group.

In addition to assessing inflammatory response and apoptotic progression, we measured the concentrations of SOD, MDA, GSH, and ROS to evaluate the extent of oxidative stress in the heart. The results (Figures 4A–D) revealed a significant decrease in the antioxidant biomarkers SOD and GSH in the CLP group, while these biomarkers were markedly elevated in the EVO group. Conversely, oxidative stress markers such as MDA and ROS showed the opposite trend. Given their close association with oxidative reactions, ATP levels were also measured to assess the energy status of the heart. As shown in Figure 4E, ATP levels were significantly reduced in the CLP group but substantially increased in the EVO group. These findings collectively indicate that EVO treatment significantly mitigates sepsis-induced myocardial oxidative stress and preserves cardiac energy metabolism.

To further validate this hypothesis, we examined mitochondrial morphology in heart tissue using TEM to observe changes in mitochondrial structure in conjunction to oxidative stress. As depicted in Figure 4F, mitochondria (yellow arrows) in the Sham group were organized in rows between the myofibrils, exhibiting well-defined double membranes, a smooth outer membrane, and a homogeneous matrix. The cristae were densely packed, uniformly organized, and distinct. However, in the CLP group, mitochondria (red arrows) were swollen, fragmented, and disordered, accompanied by ruptured myocardial fibers and prominent vacuolations. These abnormalities were attenuated in the EVO group. Additionally, we observed more frequent mitochondrial fission and fusion events (green arrows) in the EVO group compared to the Sham and CLP groups. To further assess this trend, we assessed the protein expression of biomarkers associated with mitochondrial fission and fusion in mice heart tissue. Western blot analysis (Figures 4G–I) showed that while Drp1 and Mfn2 expression levels were elevated in both the CLP and EVO groups, the increase was more pronounced in the EVO group compared to the CLP group. Collectively, these results suggest that EVO treatment effectively protects against sepsis-induced mitochondrial structural damage and actively enhances mitochondrial dynamics in the septic heart.

### 3.5 PCSK9 inhibitor promotes mitophagy via PINK1/Parkin pathway in mice with SIMD and facilitates mitophagic flux in LPS-challenged H9c2 cells

**FIGURE 5:**
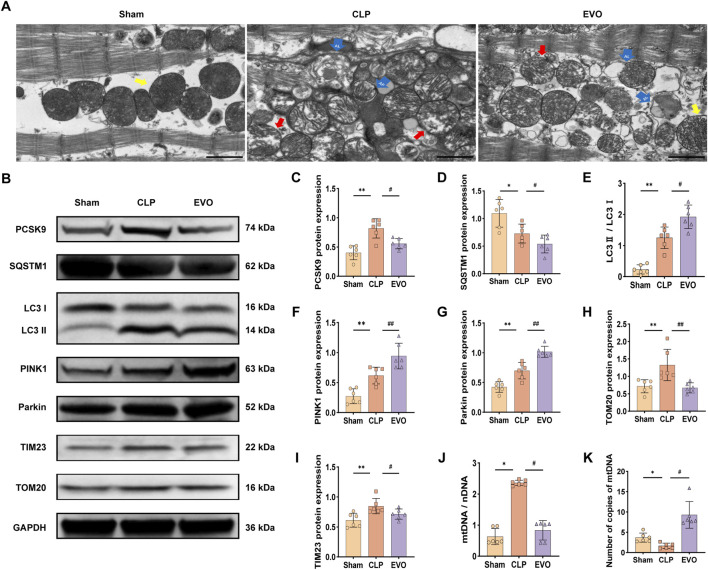
PCSK9 inhibitor improves mitophagy via PINK1/Parkin pathway in heart of mice with SIMD. **(A)** Representative images of cardiac TEM (scale bar = 1 μm, magnification: 10000×), yellow arrows indicate normal mitochondria, red arrows denote abnormal mitochondria (e.g., swollen with disrupted cristae), and blue arrows represent autophagosomes (AP) or autolysosomes (AL). **(B)** Illustrative Western blot images of key biomarkers during mitophagy process. **(C)** Quantification of PCSK9 **(D)** SQSTM1 **(E)** LC3 **(F)** PINK1 **(G)** Parkin **(H)** TOM20 and **(I)** TIM23 in Western blot images. **(J)** Statistical assessment of mtDNA to nDNA ratio and **(K)** the copies number of mtDNA using qPCR assay. N = 6 per group. *, p < 0.05 and **, p < 0.01 versus Sham group. #, p < 0.05 and ##, p < 0.01 versus CLP group.

Using TEM to examine mitochondrial morphology in the hearts of mice, we also observed that mitophagic process (blue arrows) occurred frequently in both the CLP and EVO groups (Figure 5A). To further investigate the mitophagy process, Western blot analysis was performed to evaluate the expression of key mitophagy biomarkers. The results (Figures 5B–I) revealed that EVO effectively reduced the elevated expression of PCSK9 in the CLP group. Additionally, a progressive increase in the mitophagy-related protein LC3 was observed in both the CLP and EVO groups compared to the Sham group. This increase was accompanied by an upregulation of mitophagy-associated signaling proteins, such as PINK1 and Parkin. Conversely, the protein level of the mitophagy substrate SQSTM1 significantly decreased. Since SQSTM1 is continuously degraded during autophagic clearance, this reduction is biologically consistent with the upregulation of other mitophagy-related proteins, collectively indicating an active mitochondrial turnover balance. Furthermore, the expression levels of the mitochondrial resident proteins TIM23 and TOM20 were significantly elevated in the CLP group relative to the Sham group, reflecting an accumulation of damaged mitochondria due to impaired clearance. Importantly, EVO treatment effectively reversed this accumulation, further confirming the restoration of mitophagic activity. Additionally, mtDNA/nDNA ratio and the number of mtDNA copies were assessed as indicators of mitochondrial quality and mitophagy progression. The results (Figures 5J,K) revealed that while the mtDNA/nDNA ratio was significantly elevated in the CLP group, EVO treatment effectively reduced it to near Sham group levels; conversely, the number of mtDNA copies exhibited a completely opposite trend. Taken together, these findings suggest that although mitophagy was progressively promoted in both the CLP and EVO groups, mitochondrial quality in the EVO group was superior to that in the CLP group, and mitophagy activity appeared to be somewhat restrained in the CLP group.

To further explore whether EVO might promote autophagic flux rather than merely altering static protein expression or mitochondrial mass, an *in vitro* autophagic flux assay was performed using LPS-challenged H9c2 cells. CQ was utilized during the final 6 h to blockade autophagosome-lysosome fusion (Supplementary Figure S1A). Compared to the LPS group, EVO treatment appeared to facilitate the clearance of the autophagic substrate SQSTM1 while maintaining relatively high levels of LC3II (Supplementary Figure S1B,E). Importantly, the co-administration of CQ with EVO (LPS + EVO + CQ group) led to a more pronounced accumulation of both LC3-II and SQSTM1. By quantifying the net accumulation or clearance (ΔFlux), we observed that the rate of autophagic flux was likely enhanced in the EVO-treated group compared to the LPS model (Supplementary Figure S1F). Furthermore, we monitored the mitochondrial outer membrane protein TOM20 to specifically assess mitophagic flux. While EVO attenuated the LPS-induced accumulation of TOM20, the addition of CQ largely reversed this effect, resulting in noticeable TOM20 accumulation (Supplementary Figure S1C). The quantitative ΔFlux analysis of TOM20 suggested a potentially accelerated turnover rate (Supplementary Figure S1F). Collectively, these dynamic protein alterations suggest that the EVO-mediated reduction in TOM20 may be associated with enhanced mitophagic clearance, which could contribute to the restoration of mitochondrial quality control.

### 3.6 Mdivi-1 aggravates mitochondrial damage and oxidative stress in mice with SIMD following PCSK9 inhibitor administration

**FIGURE 6:**
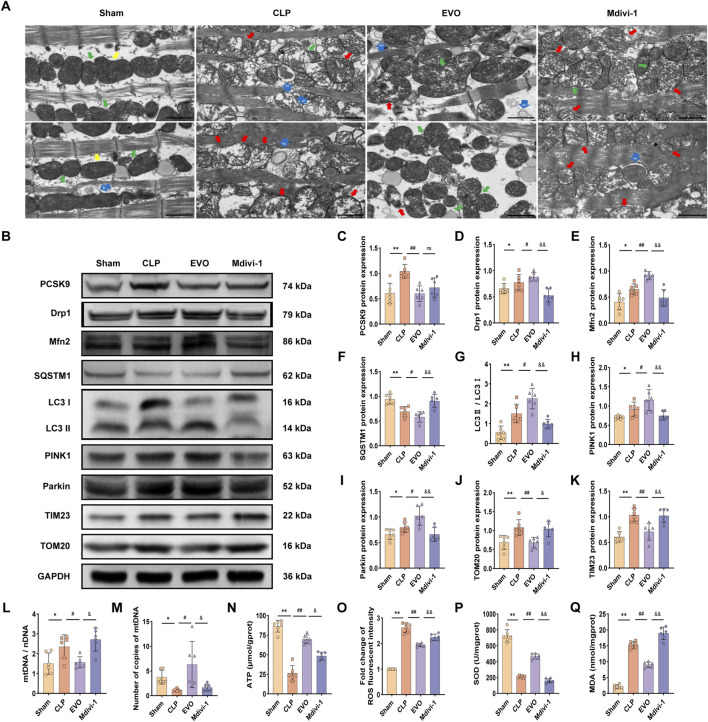
Inhibition of mitophagy aggregates mitochondrial damage and oxidative stress in mice with SIMD after PCSK9 blockage. **(A)** Representative cardiac TEM snapshots (scale bar = 1 μm, magnification: 10000×). Yellow arrows indicate normal mitochondria, red arrows denote abnormal mitochondria (e.g., swollen with disrupted cristae), green arrows highlight mitochondrial dynamics (fission or fusion) and blue arrows represent autophagosomes (AP) or autolysosomes (AL). **(B)** Illustrative Western blot images towards typical biomarkers of mitophagy. **(C)** Quantification of PCSK9 **(D)** Drp1 **(E)** Mfn2 **(F)** SQSTM1 **(G)** LC3 **(H)** PINK1 **(I)** Parkin **(J)** TOM20 and **(K)** TIM23 in Western blot images **(L)** mtDNA to nDNA ratio and **(M)** copies number of mtDNA were measured via qPCR **(N)** ATP **(O)** ROS **(P)** SOD and **(Q)** MDA concentrations in hearts were detected using colorimetric analysis. N = 6 per group. Ns, not significant. *, p < 0.05 and **, p < 0.01 versus Sham group. #, p < 0.05 and ##, p < 0.01 versus CLP group. and, p < 0.05 and andand, p < 0.01 versus EVO group.

To further investigate whether mitophagy is dependent on other phenotypes, Mdivi-1, a mitochondrial division inhibitor that consequently impairs mitophagy, was used in subsequent experiments. TEM images (Figure 6A) revealed that while the spatial localization of mitochondria in the Mdivi-1-treated group—distributed along the myocardial fibers—was similar to that observed in the other groups (Figures 4F, 5A), their ultrastructural morphology was markedly compromised. Specifically, the severe mitochondrial damage induced by Mdivi-1 closely resembled the pathological pattern of the CLP group. Mitochondria (red arrows) in the Mdivi-1-treated group became swollen, fragmented, and disordered. Additionally, the frequency of mitochondrial dynamics events (green arrows), such as fission and fusion, was lower in the Mdivi-1 group compared to the CLP and EVO groups. Western blot analysis was performed to assess the expression levels of relevant biomarkers. The results (Figures 6B–K) revealed that, following Mdivi-1 treatment, the inhibition of PCSK9 remained unchanged, whereas the expression levels of all other biomarkers were counteracted and reverted to levels comparable to those in the CLP group. A similar trend was observed in the mtDNA/nDNA ratio and the number of mtDNA copies (Figures 6L,M). Moreover, based on these mitochondrial alterations, oxidative stress in heart tissue from the Mdivi-1 group was assessed. The results (Figures 6N–Q) revealed a similar pattern, with Mdivi-1 treatment reversing the attenuation of oxidative stress observed with the PCSK9 inhibitor.

### 3.7 Mdivi-1 deteriorates inflammatory response, apoptotic process and cardiac dysfunction in mice with SIMD following PCSK9 inhibitor treatment

**FIGURE 7:**
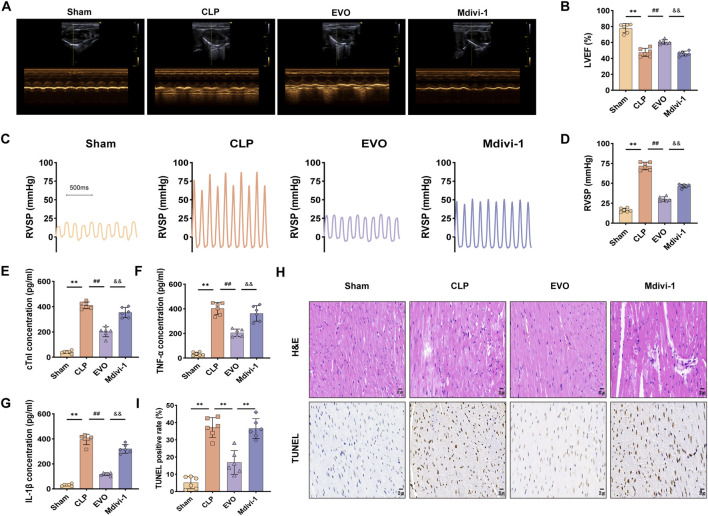
Inhibition of mitophagy deteriorates cardiac dysfunction, injury, inflammation and apoptosis in mice with SIMD following PCSK9 blockage. **(A)** Representative echocardiography snapshots towards left ventricle. **(B)** Quantification of LVEF in echocardiography snapshots. **(C)** Typical RVSP curve across different groups. **(D)** Statistical analysis of RVSP **(E)** Serum cTnI **(F)** TNF-α and **(G)** IL-1β concentrations were measured by colorimetry assay. **(H)** Illustrative histopathological and TUNEL immunohistochemical images of heart tissue (scale bar = 20 μm, magnification: ×40). **(I)** Quantitative assessment to apoptosis positive rate. N = 6 per group. **, p < 0.01 versus Sham group. ##, p < 0.01 versus CLP group. andand, p < 0.01 versus EVO group.

Based on the results shown in Figures 2, 3, we further investigated the role of mitophagy in inflammation, apoptosis, and cardiac dysfunction. The data (Figures 7A–D) indicated that LVEF and RVSP, representing left ventricular systolic performance and right ventricular pressure load respectively, both significantly reverted to levels observed in the CLP group after Mdivi-1 intervention, compared to the EVO group. Figure 7E revealed more severe cardiac injury in the Mdivi-1 group than in the EVO group. Additionally, the levels of inflammatory cytokines (Figures 7F,G) were significantly higher in the Mdivi-1 group, indicating a more pronounced inflammatory response. H&E staining (Figure 7H) further corroborated these findings, revealing disorganized and swollen myocardial tissue infiltrated by inflammatory cells. Furthermore, TUNEL immunohistochemistry and apoptosis analysis (Figures 7H,I) showed a more advanced apoptotic process in the Mdivi-1 group compared to the EVO group.

### 3.8 Mdivi-1 exacerbates cell injury, inflammatory response, cellular apoptosis and mitochondrial dynamics in LPS-administered HL-1 cells following PCSK9 inhibition

**FIGURE 8:**
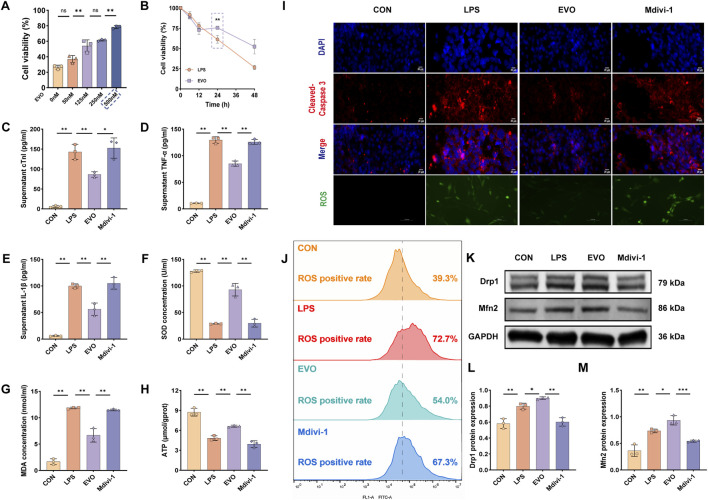
Mitophagy inhibitor exacerbates cellular injury, inflammation, oxidative stress and mitochondrial damage via restraining mitochondrial dynamics in HL-1 cells. **(A)** Viability of HL-1 cells with varying PCSK9 inhibitor concentrations and **(B)** over a time gradient were measured by CCK8 assay. The dashed boxes indicate the EVO concentrations and intervention times selected for subsequent cellular experiments. **(C)** Concentration of cTnI **(D)** TNF-α **(E)** IL-1β **(F)** SOD and **(G)** MDA in supernatant were captured using colorimetric assay. **(H)** Quantitative analysis of ATP level in cells. **(I)** Representative fluorescent images of cleaved-caspase 3 (scale bar = 20 μm, magnification: ×40) and ROS (scale bar = 100 μm, magnification: ×20) in HL-1 cells. **(J)** Illustrative flow cytometry plots of ROS in HL-1 cells. **(K)** Typical Western blot snapshots of Drp1 and Mfn2. **(L)** Quantitative analysis of Drp1 and **(M)** Mfn2 expression level in Western blot images. N = 3 per group. Ns, not significant. *, p < 0.05; **, p < 0.01 and ***, p < 0.001.

Further investigations were conducted in HL-1 cells to explore the complex interactions between PCSK9, mitophagy, and other pathophysiological phenotypes. Figures 8A–C revealed that EVO most effectively enhanced cell viability at a concentration of 500 nM with a 24-h treatment duration. Furthermore, although PCSK9 inhibition significantly improved cell viability and reduced cell injury following LPS treatment, these protective effects were reversed by Mdivi-1. The results from the inflammatory response (Figures 8D,E) and oxidative stress assays (Figures 8F,G) revealed that Mdivi-1 significantly aggravated both processes following PCSK9 inhibition. ATP measurements (Figure 8H) showed a more severe mitochondrial damage in the Mdivi-1 group compared to the EVO group, indicating the detrimental impact of Mdivi-1 on mitochondrial function after PCSK9 inhibition. Additionally, fluorescence imaging and flow cytometry (Figures 8I,J) showed a significant decrease in key markers of apoptosis and oxidative stress in the EVO group, which were notably reversed following Mdivi-1 treatment in HL-1 cells. Furthermore, Western blot analysis of mitochondrial fission and fusion markers was performed to assess mitochondrial dynamics. The results (Figures 8K,M) revealed that, while Drp1 and Mfn2 expression was progressively increased following LPS and EVO treatment, Mdivi-1 significantly inhibited the expression of both Drp1 and Mfn2. Taken together, these findings suggest that PCSK9 inhibition has beneficial effects on cellular injury, inflammation, and apoptosis in HL-1 cells; however, Mdivi-1 exacerbates and even reverses these effects.

### 3.9 Mdivi-1 reverses the enhancing effect of PCSK9 inhibitor on mitophagy via the PINK1/Parkin pathway and worsens mitochondrial damage in LPS-treated HL-1 cells

**FIGURE 9:**
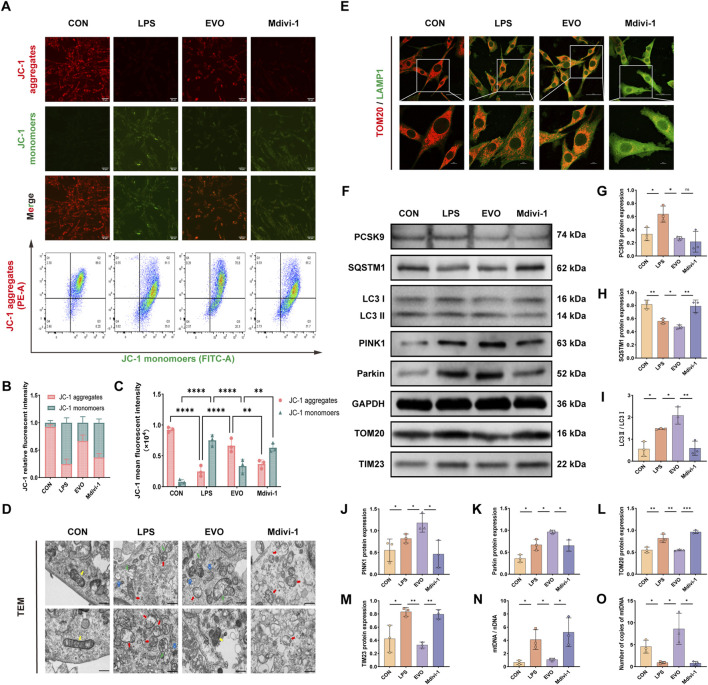
Mdivi-1 reverses enhancement of PINK1/Parkin-induced mitophagy in HL-1 cells following subsequent to PSCK9 inhibition. **(A)** Representative fluorescent images (scale bar = 50 μm, magnification: ×20) and flow cytometry plots of JC-1 in cells. **(B)** Quantification of JC-1 relative fluorescent intensity in fluorescent images. **(C)** Quantification of JC-1 mean fluorescent intensity in flow cytometry plots. **(D)** Typical TEM snapshots of HL-1 cells (scale bar = 500 nm, magnification: 20000×). Yellow arrows indicate normal mitochondria, red arrows denote abnormal mitochondria (e.g., swollen with disrupted cristae), green arrows highlight mitochondrial dynamics (fission or fusion) and blue arrows represent autophagosomes (AP), autolysosomes (AL) or phagophores (PP). **(E)** Illustrative immunofluorescent confocal images of TOM20 and LAMP1 in cells (scale bar = 50 μm, magnification: 60×, zoomed scale bar = 10 μm, zoomed maginification: 120×). **(F)** Representative Western blot images of key mitophagy biomarkers in cells. **(G)** Quantitative analysis of PCSK9 **(H)** SQSTM1 **(I)** LC3 **(J)** PINK1 **(K)** Parkin **(L)** TOM20 and **(M)** TIM23 expression level in Western blot images **(N)** mtDNA to nDNA ratio and **(O)** copies number of mtDNA in HL-1 cells were detected by qPCR. n = 3 per group. Ns, not significant. *, p < 0.05, **, p < 0.01, ***, p < 0.001 and ****, p < 0.0001.

To further investigate the role of PCSK9 in mitophagy, we conducted a more detailed examination of different phases of mitophagy in HL-1 cells. JC-1 staining, a measure of MMP, was performed using both fluorescent imaging and flow cytometry to assess the early stage of mitophagy. The results (Figures 9A–C) showed that, compared to LPS-treated cells, PCSK9 inhibition significantly alleviated mitochondrial damage, consistent with the findings presented in Figure 8G. However, the administration of Mdivi-1 reversed this effect, intensifying MMP polarization and leading to more severe mitochondrial damage.

TEM was used to examine the mid-stage of mitophagy. The images (Figure 9D) revealed that mitochondria (yellow arrows) in the control group appeared as oval or cylindrical structures with a smooth double membrane, distinct cristae, and a well-defined matrix. In contrast, mitochondria (red arrows) in LPS-treated HL-1 cells were swollen, ruptured, and damaged. Although mitophagic process (blue arrows), mitochondrial fission and fusion (green arrows) were observed, the mitochondrial repair mechanisms and quality control processes could not restore balance. PCSK9 inhibition reduced mitochondrial damage, promoted more frequent mitochondrial dynamics, and enhanced mitophagy. However, Mdivi-1 reversed the beneficial effects of PCSK9 inhibition on mitochondrial repair.

Furthermore, the late stage of mitophagy was analyzed by confocal microscopy, detecting TOM20 and LAMP1, which are key markers of mitochondria and lysosomes, respectively. Fluorescence imaging (Figure 9E) showed that mitochondria in the control group were arranged in an orderly network and tubular structure. In LPS-treated HL-1 cells, mitochondria began to obviously merge with lysosomes, and the intensity of the merged fluorescence was stronger after PCSK9 inhibitor treatment. However, Mdivi-1 treatment led to a significant increase in LAMP1 expression, and mitochondria were scarcely detectable, indicating that Mdivi-1 not only impaired mitochondrial quality control but also exacerbated cellular apoptosis.

Additionally, Western blot analysis and mtDNA measurements were used to assess the final stage of mitophagy. The results (Figures 9F–K) revealed that PCSK9 expression was significantly elevated following LPS treatment but was markedly suppressed by EVO. Furthermore, PCSK9 inhibition significantly increased the expression of mitophagy-related proteins, including LC3, PINK1, and Parkin, while reducing the mitophagy substrate SQSTM1 in comparison to LPS-treated HL-1 cells. In contrast, Mdivi-1 blocked and reversed these effects. The expression of TOM20 and TIM23, along with the mtDNA/nDNA ratio (Figures 9L–O), was measured to assess mitochondrial mass and overall turnover balance. PCSK9 inhibition restored mitochondrial mass, as evidenced by the increased steady-state levels of TOM20, TIM23, and mtDNA. However, Mdivi-1 disrupted this turnover process and caused an abnormal accumulation of TOM20, TIM23, and mtDNA. The mtDNA copy number (Figure 9P) indicated that overall mitochondrial quality control was enhanced after PCSK9 inhibition, but Mdivi-1 reversed this effect by impairing mitochondrial clearance mechanisms.

Taken together, these findings suggest that PCSK9 inhibition enhances mitophagy via the PINK1/Parkin pathway to ameliorate mitochondrial damage, and this enhancement can be blocked and reversed by Mdivi-1, a mitophagy inhibitor.

## 4 Discussion

Sepsis carries a highly variable but substantial global burden, with mortality rates typically ranging from 15% to over 30% depending on disease severity, clinical settings, and geographical regions (Singer et al., 2026; Singer et al., 2016). SIMD is a critical complication that further exacerbates this high mortality, yet effective mitochondria-targeted therapeutic strategies remain clinically scarce (Aissaoui et al., 2025; Walley, 2018). While PCSK9 is well-established in lipid metabolism, its potential role in sepsis-related cardiac injury remains elusive (Ajoolabady et al., 2025). In the present study, our findings suggest that pharmacological inhibition of PCSK9 by EVO may exert cardioprotective effects against SIMD in both CLP-induced mouse models and LPS-stimulated cardiomyocytes. Specifically, we observed that PCSK9 expression was abnormally elevated during SIMD. Furthermore, targeted inhibition of PCSK9 was associated with the alleviation of global cardiac dysfunction, structural damage, and inflammatory storms. Mechanistically, our results imply that the protection against sepsis-induced mitochondrial dysfunction may be mediated, at least in part, by the enhancement of PINK1/Parkin-associated mitophagy, which appears to facilitate the clearance of damaged mitochondria and mitigate excessive oxidative stress and cardiomyocyte apoptosis. Collectively, these findings provide new insights into the potential lipid-independent mechanisms of PCSK9 and suggest that its inhibition could serve as a promising therapeutic strategy for septic cardiomyopathy.

Traditionally, PCSK9 is recognized for its classical role in lipid metabolism via the degradation of LDLRs (Ajoolabady et al., 2025; Agnello et al., 2024). However, emerging evidence points toward its pleiotropic, non-classical functions, particularly its involvement in modulating inflammatory responses and cell survival during critical illnesses (Seidah and Prat, 2022; Hummelgaard et al., 2023; Seidah and Prat, 2022; Hummelgaard et al., 2023; Seidah and Prat, 2022; Hummelgaard et al., 2023). For instance, previous studies have suggested that PCSK9 loss-of-function mutations or pharmacological inhibition might correlate with reduced systemic inflammation and improved clinical outcomes in sepsis patients (Magnasco et al., 2022). Despite these epidemiological clues, the specific role of PCSK9 in SIMD has remained largely unexplored. Our study provides an innovative perspective by evaluating the therapeutic potential of a well-known lipid-lowering agent in the context of SIMD. Rather than functioning solely through classical lipid clearance, our data suggest that PCSK9 may directly or indirectly participate in the regulation of mitochondrial homeostasis in cardiomyocytes. By targeting this non-classical, mitochondria-associated pathway, the administration of EVO appears to offer a novel strategy to preserve myocardial energy metabolism, thereby addressing the critical lack of targeted interventions for SIMD.

At the cellular level, the pathogenesis of SIMD involves a complex cascade of interconnected phenotypes. Our results suggest a hierarchical relationship where impaired mitochondrial quality control may serve as a critical initiating event. During the progression of SIMD, we observed a compensatory increase in mitophagic activity; however, this endogenous response appeared insufficient to clear the overwhelming accumulation of damaged mitochondria (Iba et al., 2024). The subsequent failure to remove these dysfunctional organelles is closely associated with excessive ROS production, which likely acts as an upstream trigger exacerbating downstream inflammatory storms and apoptotic signaling (Murphy and Liu, 2023). By promoting PINK1/Parkin-associated mitophagy, EVO treatment facilitated the clearance of defective mitochondria, thereby attenuating oxidative stress and potentially halting the downstream inflammatory and apoptotic cascades. Interestingly, although EVO administration significantly preserved mitochondrial structural integrity, it did not completely restore ATP levels to those observed in the Sham group. This partial energy recovery implies that while enhanced mitophagy effectively mitigates mitochondrial toxicity by removing “debris,” it may not fully compensate for the profound systemic energy substrate depletion inherent to severe sepsis, nor does it necessarily induce a proportional upregulation in mitochondrial biogenesis (Lin et al., 2020; Zhang et al., 2020).

The clinical translatability of experimental findings is a primary consideration in emergency medicine research. For *in vitro* experiments, HL-1 and H9c2 cells were treated with EVO at a working concentration of 500 nM for 24 h. This optimal concentration was determined strictly based on our preliminary dose-response cell viability screening (as presented in Figures 8A–B), which indicated maximal cytoprotection against LPS without inducing basal cytotoxicity. Importantly, this selected concentration aligns well with clinically relevant serum concentrations; pharmacokinetic studies have indicated that standard dosing regimens of EVO in patients typically achieve mean steady-state peak concentrations consistent with the effective dosages used in our cell models. However, the *in vivo* dosing rationale presents a distinct translational challenge. For our *in vivo* experiments, an initial EVO loading dose of 50 mg/kg was administered. Unlike human or hamster PCSK9, wild-type murine PCSK9 exhibits extremely low binding affinity for EVO (Center for Drug Evaluation and Research, 2015). Therefore, a robust dosage of 50 mg/kg was empirically selected to compensate for this cross-species pharmacological disparity, effectively neutralizing the acute surge of PCSK9 during the severe systemic inflammatory storm of the CLP model, consistent with established preclinical safety margins. However, it is also important to consider that the complex pathophysiological environment of sepsis, characterized by altered volume of distribution and organ perfusion, may influence the myocardial distribution of EVO (Serova et al., 2026). Therefore, while our data provide a promising pharmacological basis, further clinical investigations are warranted to determine the optimal dosing window and efficacy in critically ill patients.

Despite the promising findings, several limitations in our study should be acknowledged. First, our mechanistic investigations predominantly relied on pharmacological agents. For instance, while Mdivi-1 is widely utilized in literature to evaluate mitophagy through Drp1 inhibition, it is not an entirely specific mitophagy inhibitor and may possess off-target effects (Bordt et al., 2017; Smith and Gallo, 2017). Secondly, regarding the *in vitro* models, HL-1 cells are of murine atrial origin and may not completely recapitulate the precise pathophysiological and contractile features of ventricular dysfunction typical in SIMD. Although we incorporated H9c2 cardiomyocytes to specifically assess the mitophagic flux, both immortalized cell lines inherently differ from adult primary ventricular cardiomyocytes in metabolic and structural maturity. Future studies employing isolated primary adult cardiomyocytes or human induced pluripotent stem cell-derived cardiomyocytes (hiPSC-CMs) are warranted to strengthen the translational reliability of our findings (Stephanie and Vander Roest, 2025). Consequently, the lack of genetic manipulation, such as the utilization of PCSK9 or PINK1/Parkin knockout/knockin mouse models, limits the definitive validation of the proposed molecular axes. Future studies employing cardiac-specific transgenic models are required to provide direct genetic evidence. Furthermore, our *in vivo* experimental design involved the early administration of EVO following the CLP procedure. In real-world clinical practice, particularly in low-income countries or resource-limited settings where healthcare access is often delayed, patients typically present with advanced stages of sepsis (Giamarellos-Bourboulis et al., 2024; Martin-Loeches et al., 2024; Chiu and Legrand, 2021). Therefore, the exact therapeutic window of EVO remains uncertain. Whether delayed pharmacological intervention can still reverse established SIMD represents a critical direction for our future translational research.

In conclusion, our study suggests that the targeted inhibition of PCSK9 exerts substantial cardioprotective effects against SIMD by enhancing PINK1/Parkin-associated mitophagy, thereby alleviating oxidative stress, inflammation, and apoptosis. These findings highlight a novel, non-classical role for PCSK9 in mitochondrial quality control and propose its inhibition as a potential adjunctive therapeutic strategy for sepsis-induced cardiac injury.

## Data Availability

The original contributions presented in the study are included in the article/supplementary material, further inquiries can be directed to the corresponding author.
