# Supplementary material for: PCSK9 inhibition alleviates sepsis-induced myocardial dysfunction by facilitating PINK1/parkin-associated mitophagy
Source: Front Pharmacol. 2026 Jul 3;17:1844269. doi: 10.3389/fphar.2026.1844269 (PMC13375473; doi:10.3389/fphar.2026.1844269)
Supplement: Supplementary file 1 [file Table1.pdf]

Supplementary Table S1. Detailed information of primary and secondary antibodies used in this study

Antibody target	Type	Host	Application	Dilution	Manufacturer	Catalog No.
PCSK9	Primary antibody	Rabbit	IF	1:100	Proteintech	55206-1-AP
PCSK9	Primary antibody	Rabbit	WB	1:1000	Proteintech	55206-1-AP
Drp1	Primary antibody	Rabbit	WB	1:7000	Proteintech	12957-1-AP
Mfn2	Primary antibody	Rabbit	WB	1:10000	Proteintech	12186-1-AP
SQSTM1	Primary antibody	Rabbit	WB	1:8000	Proteintech	18420-1-AP
LC3	Primary antibody	Rabbit	WB	1:4000	Proteintech	14600-1-AP
PINK1	Primary antibody	Rabbit	WB	1:1000	Proteintech	23274-1-AP
Parkin	Primary antibody	Rabbit	WB	1:2000	Proteintech	14060-1-AP
TIM23	Primary antibody	Rabbit	WB	1:8000	Proteintech	11123-1-AP
TOM20	Primary antibody	Rabbit	WB	1:10000	Proteintech	11802-1-AP
GAPDH	Primary antibody	Rabbit	WB	1:8000	ZenBioScience	380626
Cleaved-Caspase 3	Primary antibody	Rabbit	IF	1:500	ServiceBio	GB11532
TOM20	Primary antibody	Rabbit	IF	1:2000	ServiceBio	GB151481
LAMP1	Primary antibody	Mouse	IF	1:200	ServiceBio	GB14104
HRP-conjugated Anti-Mouse IgG (H+L)	Secondary antibody	Goat	IF	1:500	ServiceBio	GB23301
HRP-conjugated Anti-Rabbit IgG (H+L)	Secondary antibody	Goat	IF<br>WB	1:1000	Proteintech	SA00001-2
