# Supplementary figures and images for: PCSK9 inhibition alleviates sepsis-induced myocardial dysfunction by facilitating PINK1/parkin-associated mitophagy
Source: Front Pharmacol. 2026 Jul 3;17:1844269. doi: 10.3389/fphar.2026.1844269 (PMC13375473; doi:10.3389/fphar.2026.1844269)

**A**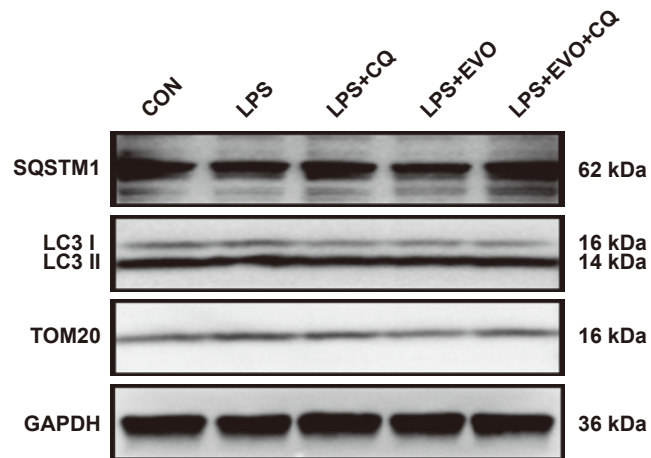**B**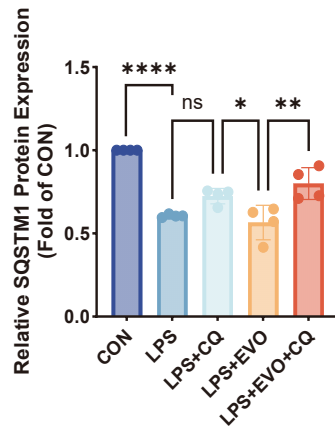**C**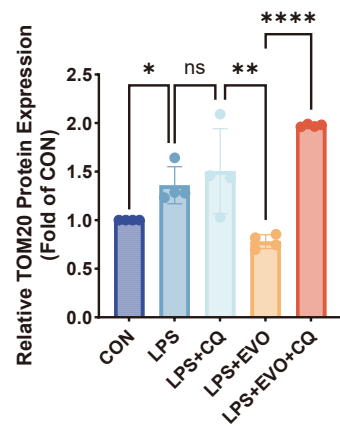**D**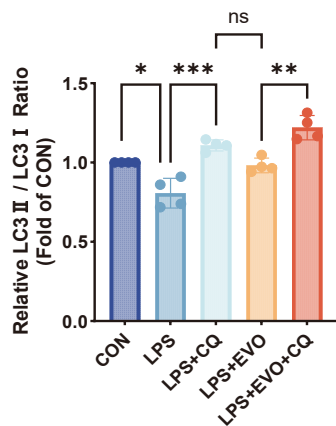**E**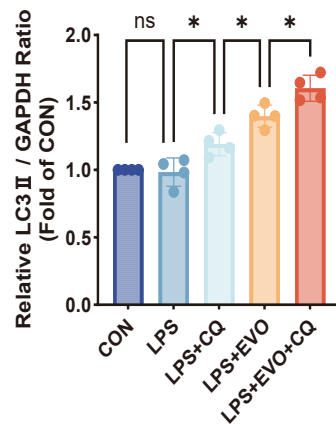**F**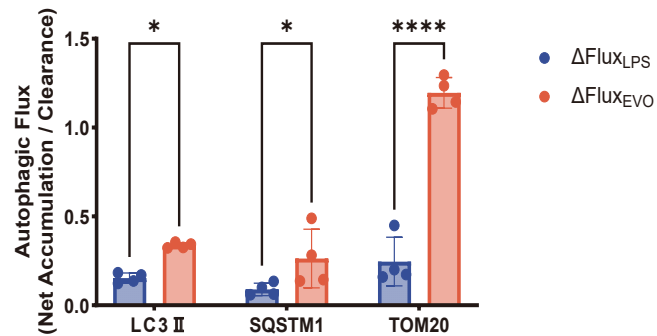

Supplement: Supplementary file 2 [file Image1.pdf]
